# Cholesterol esterification in plasma as a biomarker for liver function and prediction of mortality

**DOI:** 10.1186/s12876-017-0614-9

**Published:** 2017-04-20

**Authors:** Thorsten Kaiser, Benedict Kinny-Köster, Michael Bartels, Thomas Berg, Markus Scholz, Cornelius Engelmann, Daniel Seehofer, Susen Becker, Uta Ceglarek, Joachim Thiery

**Affiliations:** 1 Institute of Laboratory Medicine, Clinical Chemistry and Molecular Diagnostics, University Hospital Leipzig, Paul-List-Str. 13-15, 04103 Leipzig, Germany; 2 Department of Visceral, Vascular, Thoracic and Transplant Surgery, University Hospital Leipzig, Leipzig, Germany; 3 Section of Hepatology, Department of Gastroenterology and Rheumatology, University Hospital Leipzig, Leipzig, Germany; 4 Institute for Medical Informatics, Statistics and Epidemiology (IMISE), University Hospital Leipzig, Leipzig, Germany; 5 LIFE – Leipzig Research Center for Civilization Diseases, University Hospital Leipzig, Leipzig, Germany

**Keywords:** Cholesterol esterification, Mortality, Model for end-stage liver disease (MELD) score, International Normalized Ratio (INR), Orthotopic liver transplantation

## Abstract

**Background:**

Advanced stages of liver cirrhosis lead to a dramatically increased mortality. For valid identification of these patients suitable biomarkers are essential. The most important biomarkers for liver function are bilirubin and prothrombin time expressed as International Normalized Ratio (INR). However, the influence of several anticoagulants on the prothrombin time limits its diagnostic value.

Aim of this study was the evaluation of cholesterol esterification (CE) fraction (esterified cholesterol vs. total cholesterol) as an alternative biomarker for liver synthesis and mortality prediction. Under physiological conditions the CE fraction in blood is closely regulated by lecithin-cholesterol acyltransferase (LCAT) which is produced in the liver.

**Methods:**

One hundred forty-two patients with liver disease clinically considered for orthotopic liver transplant for different indications were enrolled in the study. One patient was excluded because of the intake of a direct oral factor Xa inhibitor which has a strong impact on prothrombin time.

**Results:**

Results of CE fraction were in good agreement with INR (R^2^ = 0.73; *p* < 0.001). In patients who died or survived within three months mean CE fraction was 56% vs. 74% (*p* < 0.001) and mean INR was 2.0 vs. 1.3 (*p* < 0.001), respectively. The predictive value of CE fraction for three-month mortality risk was higher compared to INR (*p* = 0.04). Results for one-year mortality were comparable.

**Conclusions:**

The cholesterol esterification fraction is a valid biomarker for liver synthesis and allows reliable prediction of mortality. In contrast to INR, it is independent of anticoagulation and other analytical limitations of coagulation tests.

**Electronic supplementary material:**

The online version of this article (doi:10.1186/s12876-017-0614-9) contains supplementary material, which is available to authorized users.

## Background

In 2013, liver cirrhosis was one of the top ten mortality causes in the developed countries [1]. In late phases of end-stage liver diseases the short-term mortality is dramatically increased. Biomarkers are essential for identification of patients with reduced liver function and increased risk for liver related mortality. For this purpose prothrombin time, cholinesterase and bilirubin are available. These parameters reflect main aspects of liver function. The prothrombin time is a global coagulation test that depends on the plasma activity of the coagulation factors X, VII, V and II. Prothrombin time respectively INR reflect mainly the capacity of the liver synthesis whereas the concentration of bilirubin is a marker for liver metabolism and the excretion of bile.

A well evaluated and widely used prognostic tool for the prediction of three-month mortality is the model of end-stage liver disease (MELD) score which is also used for organ allocation for orthotropic liver transplantation in many countries worldwide [2–4]. The MELD score is calculated from the plasma levels of creatinine, bilirubin and the prothrombin time expressed as International Normalized Ratio (INR).

However the INR as a component of the MELD score has important limitations [5–7]. Most importantly, the INR is changed by anticoagulants and substitution of coagulation factors [8–10]. It was primarily established to monitor the efficacy of the antithrombotic therapy of patients treated with vitamin k antagonists. The underlying prothrombin assays asses the global coagulation function and were not standardized for the prediction of prognosis in patients with end-stage liver disease [11, 12]. Importantly, the composition of coagulation factors in patients with end-stage liver disease differs significantly compared to patients treated with vitamin k antagonists and even in well standardized patients with oral anticoagulation these functional tests show a substantial inter-assay variability [8, 12–14].

Aim of this study was to investigate an alternative plasma biomarker to evaluate the liver function and mortality risk in patients with end-stage liver disease. We identified the cholesterol esterification (CE) fraction in blood which is very closely related to the liver function as a biomarker with potential for a translation in routine diagnostics. Under physiological conditions, cholesterol esterification is under tight control as it is essential for cellular membrane function. The most important regulatory enzyme for this regulation in blood is the lecithin-cholesterol acyltransferase (LCAT) which is produced in the liver. In fact, decreased cholesterol esterification fraction in context of liver cirrhosis has already been described in older literature [15, 16]. However, the CE fraction has not been systematically evaluated for short-term mortality prediction in patients with end-stage liver disease to date. To our knowledge, since more than 25 years no clinical studies have been published using the cholesterol esterification fraction to monitor liver function.

In this study we analyze the plasma cholesterol esterification fraction as a biomarker for liver synthesis in the context of mortality and in comparison to INR.

## Methods

We enrolled consecutively 142 patients on the liver transplantation list or in evaluation for orthotopic liver transplantation at the University Hospital Leipzig, Germany. The patients required liver transplantation for different indications (Table 1).

**Table 1:** Baseline characteristics of patients at enrollment

Number of patients	142
Patient sex	Men: 90 (63.4%)
	Women: 52 (36.6%)
Patient age (median, years)	58
Range (years)	20–77
Median MELD score	12
Range	6–40
Etiology of liver disease (including multiple nominations)	
- Alcoholic	89 (62.7%)
- Cryptogenic	19 (13.4%)
- Viral	9 (6.3%)
- NASH	9 (6.3%)
- Autoimmune hepatitis	6 (4.2%)
- PBC/PSC	2 (1.4%)
- Hepatocellular carcinoma	11 (7.7%)
Cholesterol ester	
Median (Range) [mg/dl]	154.3 (7.7–310.6)
Median (Range) [mmol/l]	3.98 (0.20–8.01)
Total cholesterol	
Median (Range) [mg/dl]	210.8 (41.3–493.7)
Median (Range) [mmol/l]	5.44 (1.07–12.74)
Cholesterol esterification fraction (median (Range))	73% (19–80%)

*PBC* Primary Biliary Cholangitis, *PSC* Primary Sclerosing Cholangitis

No additional samples for the study were taken. All measurements were performed in the residual material. After anonymization of the patients the data from the laboratory information system was analyzed retrospectively. The institutional review board of the University of Leipzig approved the study design and confirmed that an additional patient consent was not required for the measurements and the data analysis (Institutional Review Board registration number: 039-14-ff).

Medical records of all patients were retrospectively analyzed to identify potential drug interferences. One patient was excluded from further analysis, due to the intake of rivaroxaban, a new oral anticoagulation drug and another patient was lost in follow up after 268 days, due to a change of the attending transplant center. This patient was censored at this date for the analysis of one-year mortality.

### Laboratory analysis

All blood samples were taken for regular clinical laboratory diagnostics and for reporting to the European transplantation organization Eurotransplant. Creatinine and bilirubin serum levels were measured using Cobas 6000 and 8000 analyzers (Roche, Mannheim, Germany) according to the manufacturer’s instructions. Creatinine was measured using the enzymatic assay creatinine Plus Ver. 2 (Roche, Mannheim, Germany). Bilirubin was measured using the Bilirubin Total DPD Gen.2 kit (Roche, Mannheim, Germany). The prothrombin assay was performed in citrate plasma to determine the INR using an ACL TOP 700 System (Instrumentation Laboratory, Lexington, USA) with the RecombiPlasTin 2G kit (Instrumentation Laboratory, Lexington, USA).

Cholesterol esterification fraction was defined as the quotient between esterified cholesterol and total cholesterol. Total Cholesterol and cholesterol ester were analyzed in residual material of citrate plasma. For all results from citrate plasma, a dilution factor of 1.11 was applied to compensate the additional volume of citrate solution. If residual citrate plasma was unavailable, serum material was used for the analysis (53 cases). Parallel measurements (*n* = 44) were performed to assure comparability of the results. The median difference between citrate plasma and serum was 0.19% with a standard deviation of + -1.9%. Minimum and maximum difference was −4.1 and 4.6% respectively (Additional file 1: Figure S1).

Cholesterol and cholesterol ester were determined based on our previously published LC-MS/MS method [17]. In brief, 10 μl of calibrator, quality control or sample (serum or plasma) was mixed with 490 μl internal standard solution (100 μg/L d7-cholesterol in methanol/isopropanol, 1/1, v/v) into polypropylene tubes. Following centrifugation for 10 min at 11.400 g, the supernatant was transferred to glass vials for tandem mass spectrometric analysis. 25 μl of the supernatants were injected onto the analytical column (Chromolith SpeedROD RP-18e, 50 × 4.6 mm, monolithic column, Merck KGaA, Darmstadt, Germany). An API 4000 triple quadrupole mass spectrometer with an atmospheric pressure photoionization source (AB Sciex, Darmstadt, Germany) was used.

### MELD Score

MELD scores were calculated from serum creatinine (mg/dL), serum bilirubin (mg/dL) and prothrombin time (INR) according to the UNOS guidelines [18] using the following formula:

$$ 10\ \left\{0.957 \times \mathrm{Ln}\ \left(\mathrm{creatinine}\ \mathrm{mg}/\mathrm{dL}\ *\ \mathrm{A}\right) + 0.378 \times \mathrm{Ln}\ \left(\mathrm{bilirubin}\ \mathrm{mg}/\mathrm{dL}\ *\ \mathrm{B}\right) + 1.120 \times \mathrm{Ln}\ \left(\mathrm{INR}\right) + 0.643\right\} $$

$$ \mathrm{A} = 0.01131 = \left(\mathrm{creatinine}\ \mathrm{mg}/\mathrm{dL}\right)\ /\ \left(\mathrm{creatinine}\ \upmu \mathrm{mol}/\mathrm{L}\right); $$

$$ \mathrm{B} = 0.05848 = \left(\mathrm{bilirubin}\ \mathrm{mg}/\mathrm{dL}\right)\ /\ \left(\mathrm{bilirubin}\upmu \mathrm{mol}/\mathrm{L}\right) $$

According to the guidelines, creatinine (mg/dL), bilirubin (mg/dL) and INR values that were lower than 1.0 were set to 1.0 for MELD calculation. Maximum serum creatinine level was set to 4.0 mg/dL = 353.7 μmol/L. Creatinine values < 1.0 mg/dL were set to 1.0 mg/dL = 88.4 μmol/L (9).

### Statistical analysis

Data was obtained from Laboratory information management system iSOFT Laboratory version 12.8.1 (CSC, Virginia, USA). The results of measured parameters were analyzed using Excel 14.0 (Microsoft Corporation, Redmond, USA) and SPSS 22.0 (IBM, Armonk, USA). We considered three-month and one-year survival as primary endpoint, where liver transplant is counted as censoring. Predictive power of parameters on survival where analyzed via Cox-Regression. A *p* value <0.05 was considered statistically significant. Relationship of INR and CE was analyzed by linear regression. The predictive power for three-month and one-year mortality was analyzed for uncensored patients and compared using areas under the curve of the Receiver operating characteristic (AUROC). For ROC analysis and testing, the R-package “pROC” was used.

## Results

One hundred forty-two patients suffering from end-stage liver disease were included in the study. The follow-up time was one year. The intake of an oral factor Xa inhibitor (Rivaroxaban) was identified for one patient. Due to the impact on the INR, this patient was excluded from further analysis.

Ten of 141 (7%) patients died within three months and 25 of 141 (18%) within one year after study inclusion. 4 from 141 (3%) patients received a liver transplant within three months and 12 (9%) within one year.

High INR values and a low cholesterol esterification fraction were both associated with an increased three-month and one-year mortality (*p* < 0.001). The results for the cholesterol esterification fraction and INR showed a strong linear dependence (R^2^ = 0.73; *p* < 0.001).

The median cholesterol esterification fraction for patients who died within three months and one year was 56 and 65%, respectively, versus 74% in the patients who survived. See supplementary material for the quantitative results of other investigated parameters in the context of mortality, sex and age (Additional file 1: Tables S1–S4).

**Fig. 1:**
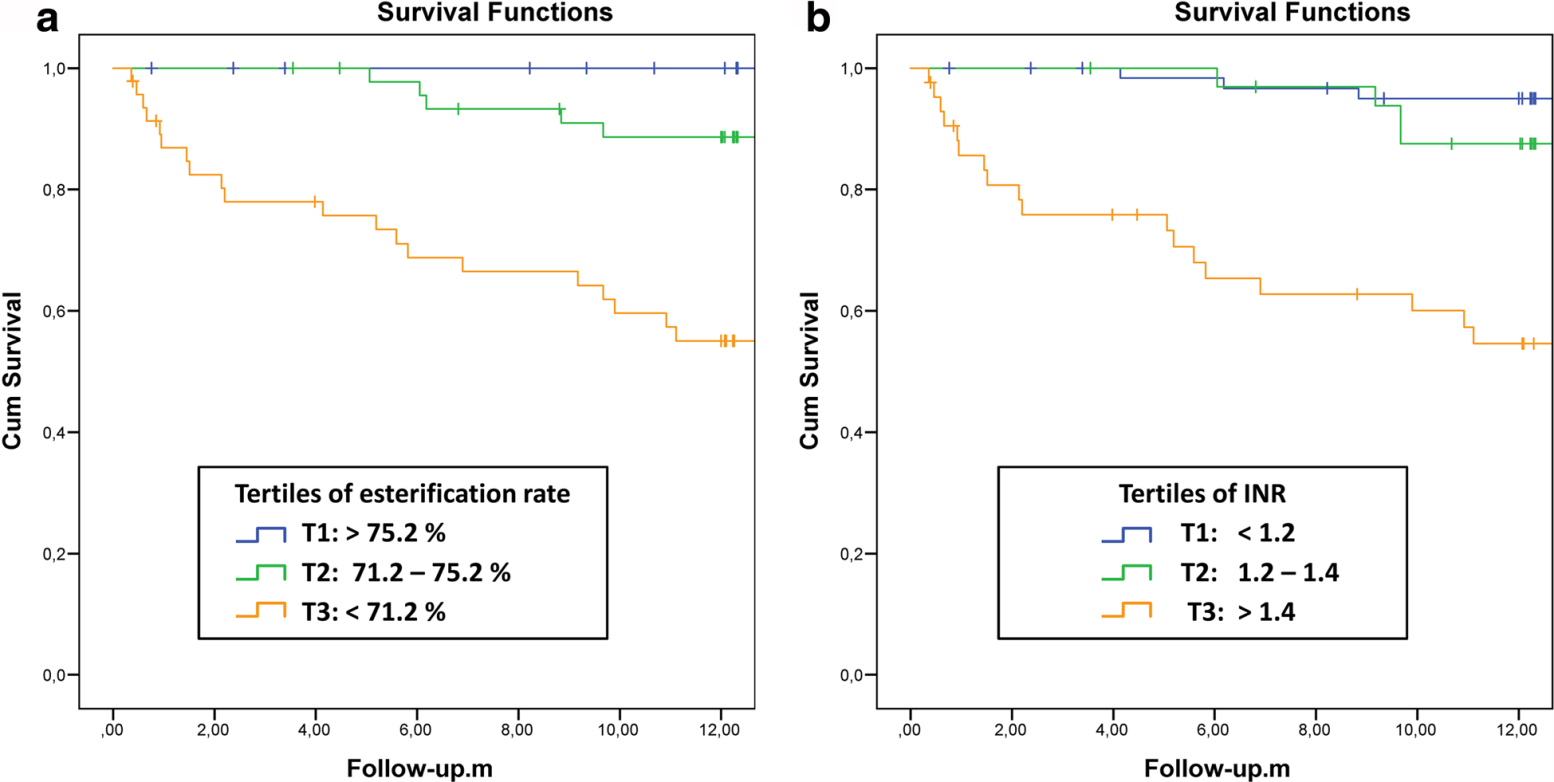
Kaplan-Meier survival analysis for tertiles according to cholesterol esterification and INR: Patients were divided in tertiles according to their **a** cholesterol esterification fraction and **b** INR. While for CE all risk groups differ significantly (Table 2), the contrast of the highest INR groups is not significant (logrank test). Censoring is indicated with vertical bars within the curves

Patients were divided in tertiles according to their cholesterol esterification rate. Next, we analyzed the association of cholesterol esterification fraction with one-year mortality rate (Fig. 1a). The same approach was used for the association of INR tertiles with mortality (Fig. 1b).

After three months 21% of the patients in the tertile with the lowest esterification fraction (<71.2%) and 23% of the patients with the highest INR tertile (>1.4) had died. None of the patients of the other tertiles had died after three months (Table 2).

**Table 2:** Comparison of three-month and one-year mortality according to cholesterol esterification fraction and INR tertiles

Parameter	Range of tertiles	3 month mortality	1 year mortality
CE-Tertiles	T1: > 75.2%	0/47 (0%)	0/47 (0%)
	T2: 71.2–75.2%	0/47 (0%)	5/47(10.6%)
	T3: < 71.2%	10/47 (21.3%)	20/47 (42.6%)
Significances for CE-Tertiles		1 vs 3 *p* < 0.001	1 vs 2 *p* < 0.023
		2 vs 3 *p* < 0.001	2 vs 3 *p* < 0.001
			1 vs 3 *p* < 0.001
INR-Tertiles	T1: < 1.2	0/64 (0%)	3/64 (4.7%)
	T2: 1.2–1.4	0/34 (0%)	4/34 (11.8%)
	T3: > 1.4	10/43 (23.3%)	18/43 (41.9%)
Significances for INR-Tertiles		1 vs 3 *p* < 0.001	1 vs 2 *p* = 0.22
		2 vs 3 *p* = 0.024	2 vs 3 *p* = 0.001
			1 vs 3 *p* < 0.001

After one year, 42.6% of the patients in the tertile with the lowest esterification fraction (<71.2%) and 10.6% of the patients from the second tertile (71.2 to 75.2%) had died. Table 2 summarizes the mortality after three months and one year according to the CE- and INR tertiles.

Interestingly, after one year none of the patients in the tertile with the highest esterification fraction (> 75.2%) had died compared to 3 patients in the INR <1.2 tertile.

**Fig. 2:**
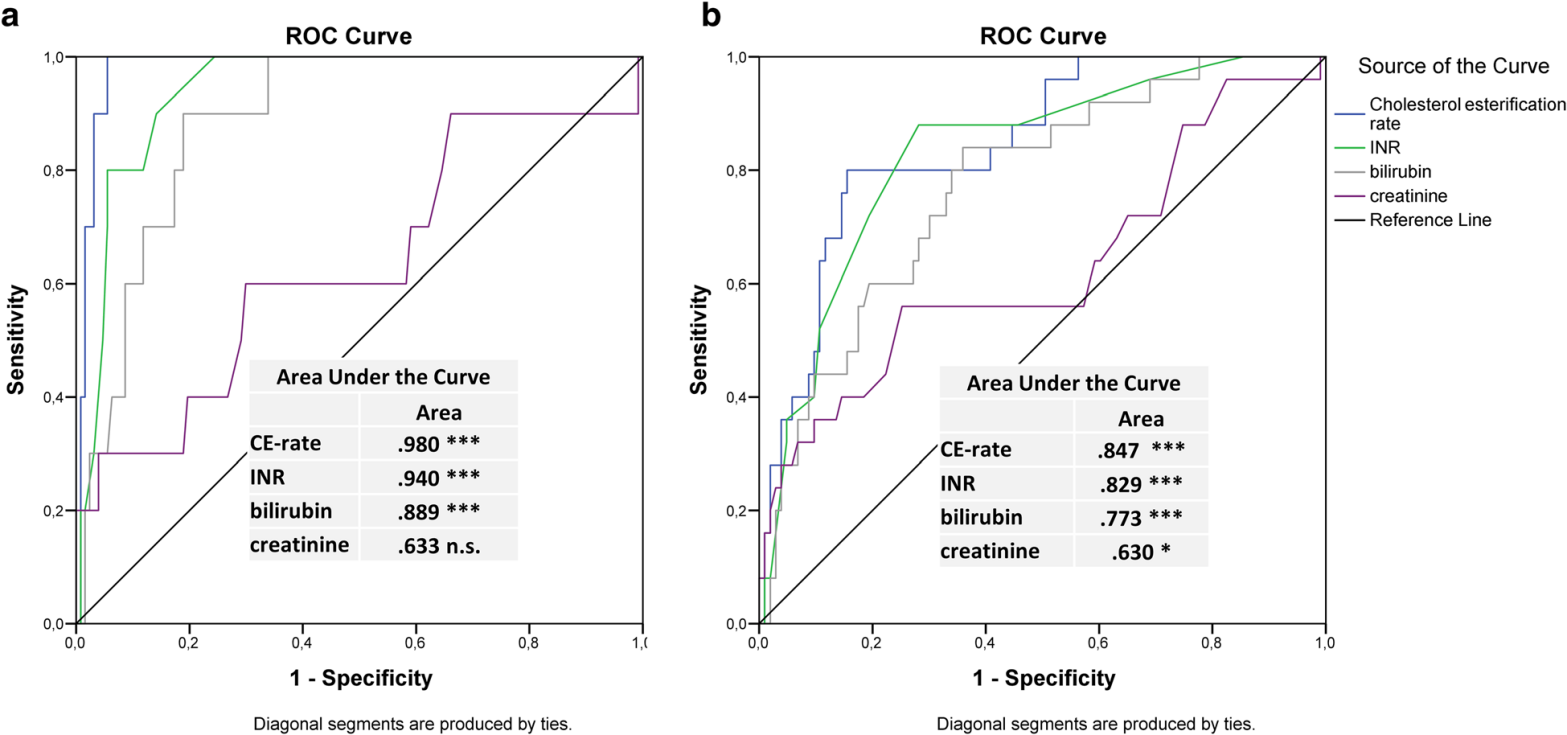
ROC analysis of single biomarkers for predicting three-month (**a**) and one-year (**b**) mortality during follow up (n.s. = not significant; * = *p* < 0.05; ** = *p* < 0.01; *** = *p* < 0.001)

We compared the receiver operating characteristic (ROC) curves of cholesterol esterification fraction and INR (Fig. 2). Area under the curve of the ROC analysis (AUROC) for the three-month mortality was 0.98 for cholesterol esterification fraction (*p* < 0.001, 95%-CI: 0.96–1.00) and 0.94 for INR (*p* < 0.001, 95%-CI: 0.89–0.99), respectively. For one-year mortality, the AUROCs were 0.85 (*p* < 0.001) and 0.83 (*p* < 0.001), respectively.

For prediction of three-month survival, ROC results of cholesterol esterification fraction were superior to those of INR (*p* = 0.04), bilirubin (*p* = 0.009) and creatinine (*p* = 0.003). INR was significantly superior to creatinine (*p* = 0.02) but not to bilirubin (*p* = 0.18).

For one-year survival Cholesterol esterification fraction and INR were both superior to creatinine (for CE *p* = 0.007, for INR *p* = 0.03), but not in comparison to each other or to bilirubin.

## Discussion

In our work, we identified the plasma cholesterol esterification fraction as a promising biomarker for the prediction of mortality in patients with end-stage liver disease. Cholesterol esterification fraction was significantly superior to INR in the prediction of three month mortality. There are several explanations for the low cholesterol esterification fraction in end-stage liver disease patients. The cholesterol esterification fraction in plasma depends mainly on the activity of the LCAT (lecithin-cholesterol acyltransferase) [19], a genetically highly conserved enzyme. LCAT is produced by the liver and secreted into the blood. The plasma concentration in persons with normal liver function show only minor variation and the half-life in plasma have been estimated to be 4–5 days [20].

LCAT has been discovered in 1962 [21] and plays an important role for the reverse cholesterol transport [22]. Genetic defects are extremely rare (<1:1,000,000) [20].

The LCAT plasma concentration may also be an interesting biomarker for liver function, but the measurement requires an immunological assay which is expensive, difficult to standardize and the result does not reflect the enzyme activity. Measurement of LCAT enzyme activity instead is complicated and not usable for clinical routine because LCAT activity is influenced by the level of ApoAI, HDL [19], sphingomyelin and phosphatidylcholine in vivo [23]. In the reaction of cholesterol esterification the abnormal lipoprotein X (LP-X) [24] has been described also. LP-X particles contain almost only unesterified cholesterol and appear typically in patients with obstructive biliary diseases and in patients with LCAT-deficiency. But so far, a quantification of LP-X for clinical routine is not available and the clinical relevance remains unclear [25].

In terms of methodical validity, the calculation of the esterification fraction from the results of plasma cholesterol ester and total cholesterol could be advantageous. The implementation of the quotient eliminates interferences with the complex matrix of samples from end-stage liver disease patients. In clinical routine cholesterol is measured on high throughput analyzers with well standardized enzymatic assays. In these assays all cholesterol esters are first hydrolyzed to free cholesterol by cholesterol ester hydrolase. The free cholesterol is then oxidized by cholesterol oxidase resulting in a chromogen which can be measured photometrically [26]. The same application without cholesterol ester hydrolase could be used in clinical routine measuring the content of free cholesterol which allows the calculation of the cholesterol ester fraction. Plasma cholesterol esterification fraction has some important advantages compared to INR as a biomarker for liver disease. Importantly, in contrast to INR cholesterol esterification fraction is not affected by anticoagulants. The patient who was excluded from further analysis in our study received an anticoagulation treatment with the direct oral factor Xa inhibitor rivaroxaban which is known to affect the INR. Consequently, in this patient the initially INR and resulting MELD score were dramatically increased. After discontinuation of the drug, INR and MELD score decreased rapidly. In contrast, the cholesterol esterification rate was almost stable (See Additional file 1 for details). Prothrombin time as well as other global coagulation tests is difficult to standardize. These assays are based on the detection of the coagulation time after activation with different thromboplastines. The characteristics of theses thromboplastines differ significantly between available assays. The results from prothrombin assays were transformed in the International Normalized Ratio (INR) using the assay specific International Sensitivity Index (ISI). However, for this standardization, exclusively samples of patients treated with vitamin k antagonists were included as monitoring of this treatment was the intention for implementation of INR. Patients taking vitamin k antagonists compared to patients suffering from liver disease show a significantly different composition of coagulation factors. Factor V, a central element of the coagulation system, is not influenced by taking vitamin k antagonists, but in patients with liver cirrhosis, it is decreased in accordance with the stage of the liver disease [27]. Consequently, the results of different assays in patients with end-stage liver disease vary significantly [28, 29]. A possible recalibration of the ISI which could eliminate some of the limitations for patients with end-stage liver disease is sophisticated and has not been implemented in clinical routine [30–33]. Consequently the laboratory diagnostics has a considerable impact on the INR and the resulting MELD score [7, 34].

For further evaluation of the cholesterol esterification fraction in the context of the other MELD parameters (bilirubin and creatinine), we used the correlation between INR and cholesterol esterification fraction to substitute INR in the MELD score. The resulting modified MELD score showed superior prediction of the three month mortality compared to the original MELD score (*p* = 0.02; see Additional file 1 for details).

Until now, the effect of antidyslipidemic drugs on the cholesterol esterification fraction has not been investigated in patients with end-stage liver disease. We analyzed the cholesterol esterification fraction in the large cohort of patients with coronary artery disease in the Leipzig (LIFE) Heart Study [35] and compared 658 patients under statins with 1213 patients without statins. The cholesterol esterification fraction did not differ relevantly between these two groups (CE rate with statins 75.4%, without 76.2%, data not shown) and was in good agreement with the cholesterol esterification fraction of the patients with ≥1 year survival in the present study.

There are some limitations of our study. The most important one is the limited number of patients and the heterogeneous causes for end-stage liver disease. Therefore, we were not able to analyze subgroups of patients in this study. If the mortality prediction is also valid for patients with hepatocellular carcinoma cannot be answered yet and will be an important question to answer in future studies.

We currently develop and evaluate an assay for quantification of free cholesterol on a high-throughput clinical chemistry analyzer system which allows, in utilization of a total cholesterol assay, the calculation of cholesterol esterification fraction. Based on this test, we plan a multicenter study to prove the diagnostic value of this rediscovered biomarker for liver function. Further research is also needed to study the metabolism of involved lipoproteins in more detail.

## Conclusions

The cholesterol esterification fraction is a valid biomarker for liver synthesis and allows reliable prediction of mortality. In contrast to INR, it is independent of anticoagulation and other analytical limitations of coagulation tests. Despite the small sample size, a benefit using the cholesterol ester fraction to estimate the liver function and to predict mortality risk in patients with livers disease in comparison with INR is plausible.

## Additional file

Additional file 1: Supplemental Data for “Cholesterol esterification in plasma as a biomarker for liver function and prediction of mortality." (PDF 610 kb)

## Abbreviations

CE: Cholesterol esterification
CE-MELD: MELD score based on the cholesterol esterification fraction
INR: International Normalized Ratio
ISI: International Sensitivity Index
LCAT: Lecithin-cholesterol acyltransferase
MELD: Model for end-stage liver disease
