# Supplementary material for: Cholesterol esterification in plasma as a biomarker for liver function and prediction of mortality
Source: BMC Gastroenterol. 2017 Apr 20;17:57. doi: 10.1186/s12876-017-0614-9 (PMC5397767; doi:10.1186/s12876-017-0614-9)
Supplement: Additional file 1: — Supplemental Data for “Cholesterol esterification in plasma as a biomarker for liver function and prediction of mortality." (PDF 610 kb) [file 12876_2017_614_MOESM1_ESM.pdf]

Supplemental Data for "Cholesterol esterification in plasma as a biomarker for liver function and prediction of mortality"

Authors: Thorsten Kaiser[1]; Benedict Kinny-Köster[1]; Michael Bartels[2], Thomas Berg[3], Markus Scholz[4]; Cornelius Engelmann[3]; Daniel Seehofer[2]; Susen Becker[1,5]; Uta Ceglarek[1,5]; Joachim Thiery[1,5]

[1] Institute of Laboratory Medicine, Clinical Chemistry and Molecular Diagnostics, University Hospital Leipzig, Germany
[2] Department of Visceral, Vascular, Thoracic and Transplant Surgery, University Hospital Leipzig, Germany
[3] Section of Hepatology, Department of Gastroenterology and Rheumatology, University Hospital Leipzig, Leipzig, Germany
[4] Institute for Medical Informatics, Statistics and Epidemiology (IMISE), University Hospital Leipzig, Leipzig, Germany
[5] LIFE – Leipzig Research Center for Civilization Diseases, University Hospital Leipzig, Germany

Corresponding Author:

Dr. med. Thorsten Kaiser

Institute of Laboratory Medicine, Clinical Chemistry and Molecular Diagnostics

University Hospital Leipzig

Paul-List-Str. 13-15

04103 Leipzig

Germany

Telephone: +49 341 9722200

Fax: +49 341 22209

E-mail: thorsten.kaiser@medizin.uni-leipzig.de

Table of contents

Comparison of laboratory results according to 3–month and 1-year-mortality 3
Details of clinical significance of CE in context of sex and aging effect 5
Comparison of CE fraction measured in serum vs. citrate plasma 7
Calculation of the CE-MELD 8
Substitution of the INR in the MELD score by the cholesterol esterification rate 9
Correlation between the original MELD score and the CE based MELD score 11
Characteristics of the patient who was excluded from further analysis after the intake of an oral factor Xa inhibitor was proven 12

Comparison of laboratory results according to 3–month and 1-year-mortality

Tables S1 and S2 summarize the laboratory parameters according to three-month and one-year mortality.

3-month mortality		Patients age [days] n.s.	Bilirubin [µmol/l] ***	Creatinine [µmol/l] n.s.	INR ***	MELD ***	MELDNa ***	CE-MELD ***	Sodium [mmol/l] *	Free cholesterol [mg/l] *	Cholesterol ester [mg/l] ***	Total cholesterol [mg/l] ***	CE-rate ***
**No**	Median	21027	28	76	1.3	11	14	11	138	540	1575	2144	0.74
	Mean	20550	43	83	1.3	13	15	12	137	593	1571	2163	0.72
	N	127	127	127	127	127	126	127	126	127	127	127	127
	Std. Deviation	3360	48	27	0.3	5	5	5	4	245	490	686	0.06
**Yes**	Median	21347	105	94	2.0	23	26	26	134	338	472	810	0.56
	Mean	20645	121	124	2.1	24	27	26	133	464	586	1050	0.54
	N	10	10	10	10	10	9	10	9	10	10	10	10
	Std. Deviation	4243	72	88	0.5	5	3	5	9	276	402	674	0.05
**total**	Median	21079	29	77	1.3	12	14	11	138	536	1547	2115	0.73
	Mean	20557	48	86	1.4	13	15	13	137	583	1499	2082	0.71
	N	137	137	137	137	137	135	137	135	137	137	137	137
	Std. Deviation	3413	54	36	0.4	6	6	6	4	248	547	742	0.08

Table S1: Laboratory parameters according to three-month mortality: sodium levels sodium levels were unavailable in two cases. Four patients were censored because of liver transplantation within three month. Significance was calculated using independent samples Mann-Whitney-U-test (n.s. = not significant; * = p < 0.05; **= p < 0.01; ***= p < 0.001).

1-year mortality		Patients age [days]	Bilirubin [µmol/l]	Creatinine [µmol/l]	INR	MELD	MELDNa	CE-MELD	Sodium [mmol/l]	Free cholesterol [mg/l]	Cholesterol ester [mg/l]	Total cholesterol [mg/l]	CE-rate
		n.s.	***	*	***	***	***	***	*	n.s.	***	***	***
**no**	Median	20779	27	75	1.2	11	13	11	138	553	1626	2182	0.74
	Mean	20356	40	79	1.3	12	14	12	137	599	1614	2213	0.73
	N	103	103	103	103	103	103	103	103	103	103	103	103
	Std. Deviation	3414	49	24	0.3	4	5	5	4	250	488	689	0.06
**yes**	Median	21083	52	94	1.6	20	23	19	136	497	998	1531	0.65
	Mean	20704	83	107	1.7	19	22	20	135	548	1035	1582	0.63
	N	25	25	25	25	25	23	25	23	25	25	25	25
	Std. Deviation	3599	65	62	0.5	6	6	7	6	272	594	838	0.09
**total**	Median	20864	29	76	1.3	12	14	11	138	543	1555	2122	0.73
	Mean	20424	49	85	1.4	13	15	13	137	589	1501	2090	0.71
	N	128	128	128	128	128	126	128	126	128	128	128	128
	Std. Deviation	3439	55	36	0.4	6	6	6	4	254	558	760	0.08

Table S2: Laboratory parameters according to 1-year mortality: sodium levels were unavailable in two cases. 13 patients were censored because of liver transplantation within one year. Significance was calculated using independent samples Mann-Whitney-U-test (n.s. = not significant; * = p < 0.05; **= p < 0.01; ***= p < 0.001).

Details of clinical significance of CE in context of sex and aging effect

Tables S3 and S4 characterize CE fraction and other relevant parameters in the context of sex and aging.

	Sex										
	Women					Men					
	Mean	Median	Minimum	Maximum	n	Mean	Median	Minimum	Maximum	n	p value
**bilirubin [µmol/L]**	57.0	29.0	4.5	346.6	52	48.4	29.4	5.7	336.3	89	0.625
**creatinine [µmol/L]**	82	69	44	313	52	91	80	41	329	89	0.011
**INR**	1.5	1.3	.9	3.4	52	1.4	1.3	.9	4.3	89	0.640
**sodium [mmol/l]**	136.9	137.7	125.8	151.0	52	136.6	137.7	117.0	145.6	89	0.930
**MELD**	13.9	11.5	6.4	39.6	52	13.6	11.8	6.4	37.2	89	0.601
**MELDNa**	15.7	14.0	6.0	40.0	52	15.5	14.2	6.0	31.3	89	0.793
**total cholesterol [mg/dl]**	230.6	231.2	46.9	493.7	52	191.6	190.2	41.3	294.7	89	0.003
**cholesterol ester [mg/dl]**	164.3	174.6	17.0	310.6	52	138.7	144.3	7.7	222.1	89	0.003
**Cholesterol esterification fraction**	.69	.72	.32	.80	52	.71	.74	.19	.80	89	0.269

Table S3: Result of parameters according to sex of the patients.

	Group according to the median age of 57.7 years										
	age < medium					age ≥ medium					
	Mean	Median	Minimum	Maximum	n	Mean	Median	Minimum	Maximum	n	p value
**bilirubin [µmol/L]**	58.7	29.5	5.7	346.6	70	44.6	29.4	4.5	300.8	71	0.923
**creatinine [µmol/L]**	79	75	41	137	70	97	79	44	329	71	0.035
**INR**	1.4	1.3	.9	4.3	70	1.4	1.3	.9	3.2	71	0.939
**sodium [mmol/l]**	136.8	137.4	125.8	143.3	70	136.7	137.9	117.0	151.0	71	0.985
**MELD**	13.4	11.6	6.4	37.2	70	13.9	12.2	6.4	39.6	71	0.583
**MELDNa**	15.2	14.0	6.0	32.0	70	15.9	15.0	6.0	40.0	71	0.714
**total cholesterol [mg/dl]**	204.5	199.7	41.3	493.7	70	207.4	212.4	44.5	436.8	71	0.726
**cholesterol ester [mg/dl]**	145.7	145.8	7.7	294.1	70	150.5	158.9	17.3	310.6	71	0.509
**Cholesterol esterification fraction**	.70	.73	.19	.80	70	.71	.73	.32	.80	71	0.740

Table S4: Comparison of the result for patients < vs ≥ median age. The median age in this cohort was 57.7 years.

Comparison of CE fraction measured in serum vs. citrate plasma

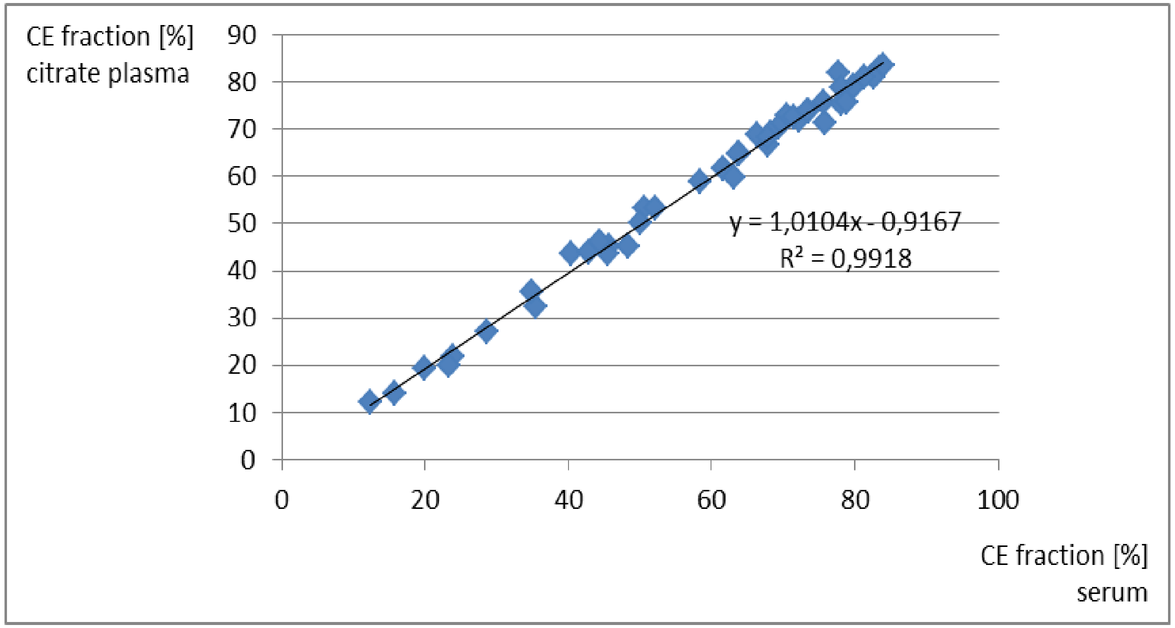

Figure S1: Comparison of CE fraction measured in citrate plasma vs. serum

Calculation of the CE-MELD

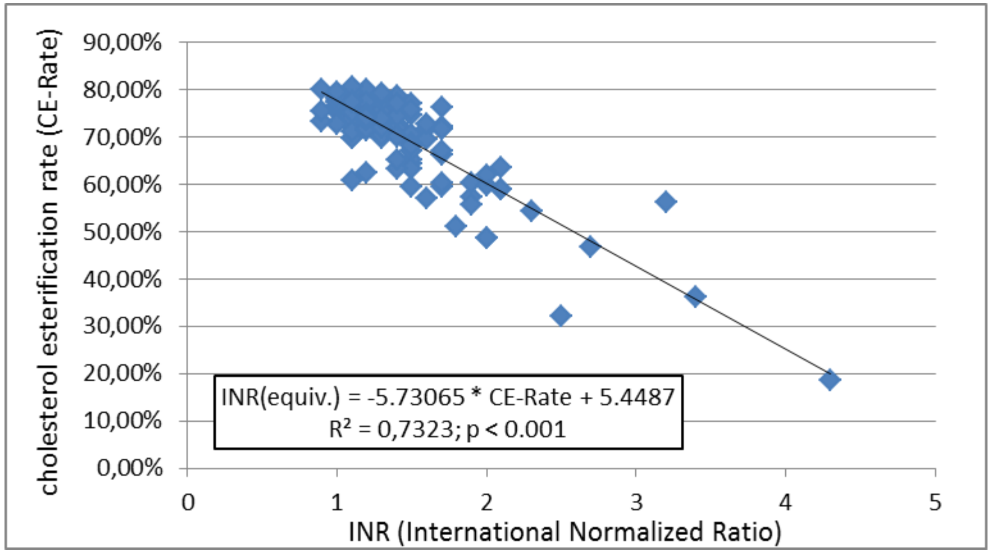

Figure S1: Correlation between cholesterol esterification rate and International Normalized Ratio (INR).

The linear correlation line followed the formula:

$$\text{cholesterol esterification rate} = -0.1745 * \text{INR} + 0.9508.$$

Substitution of the INR in the MELD score by the cholesterol esterification rate

To allow a direct comparison between the two biomarkers in the context of the MELD score and because of their close agreement, we tried to replace the INR in the MELD score with the cholesterol esterification fraction for further analysis. Based on the linear correlation curve we transformed the cholesterol esterification fraction into INR equivalents that were used for the calculation of the new CE-MELD score.

To prove the diagnostic power of the CE-MELD score in comparison to the conventional MELD score further ROC analysis was performed. For this analysis we included also the MELDNa score, which was described to have a superior diagnostic value compared to the conventional MELD score (Supplemental Figure 2).

For the prediction of three month survival CE-MELD was significantly superior to MELD ($p = 0.02$). For the prediction of one-year survival both scores did not show significant differences ($p > 0.05$).

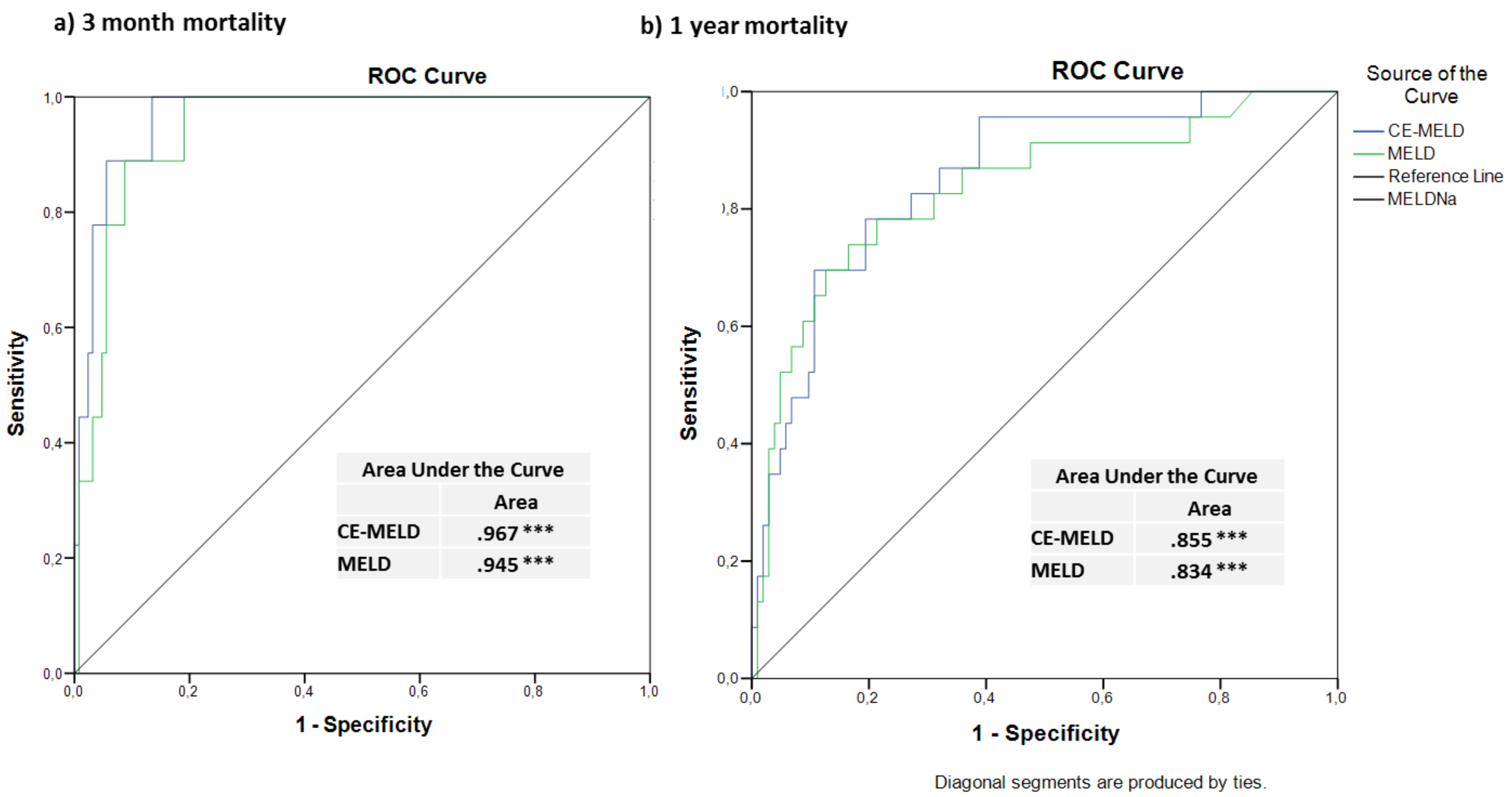

Figure S2: ROC analysis of the prognostic scores: MELD.. CE-MELD and MELDNa for three month and one-year mortality (***= p < 0.001).

Correlation between the original MELD score and the CE based MELD score.

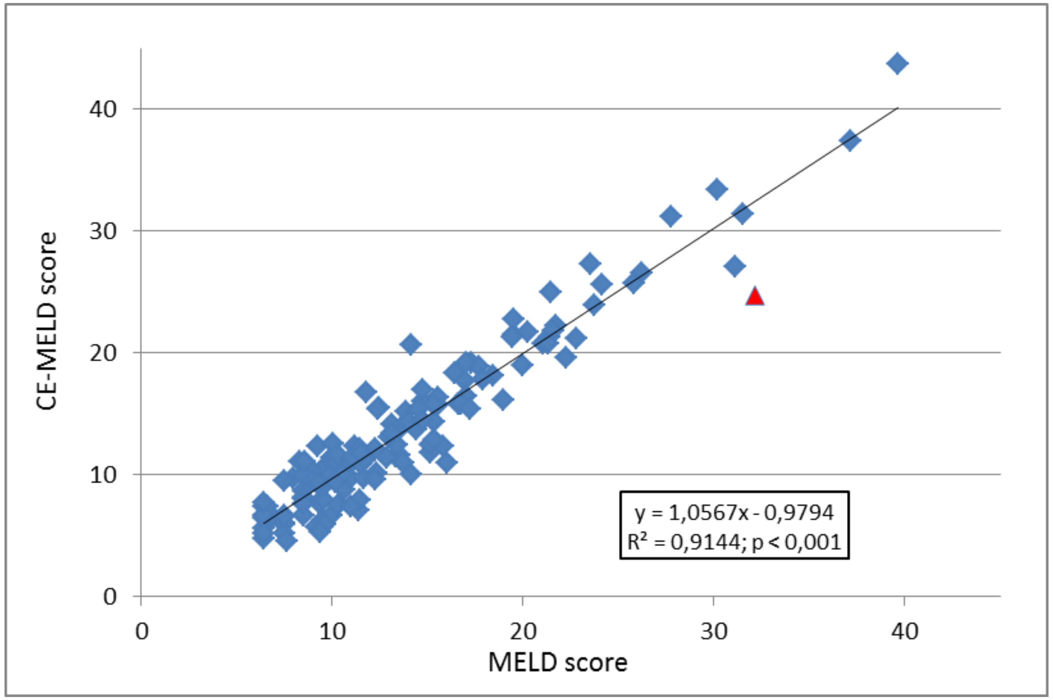

Figure S3: Resulting correlation between the original MELD score including INR and the CE-MELD score after substitution of INR. The patient who received the direct factor Xa inhibitor (rivaroxaban) prior to MELD diagnostics is marked as a red triangle. This patient was excluded from all analysis including this regression analysis.

Characteristics of the patient who was excluded from further analysis after the intake of an oral factor Xa inhibitor was proven

The patient who was excluded from further analysis after the intake of the oral factor Xa inhibitor rivaroxaban was proven suffered from an acute decompensation of chronic ethyltoxic liver cirrhosis Child B. He was ethanol abstinence for >6 months. Rivaroxaban was given for therapeutic anticoagulation because of atrial fibrillation. The patient had been initially admitted to an external hospital with an initial INR of 5.4 under anticoagulation with rivaroxaban resulting in a MELD score of 39. Because of the high MELD score and the clinical decompensation the anticoagulation was stopped and the patient was transferred to the university hospital of Leipzig for further treatment and evaluation of liver transplantation. Already on the following day after discontinuation of rivaroxaban INR dropped to 3.7 resulting in a MELD score of 32. The corresponding CE-MELD showed only 25. 18 days later the patient had recovered and could be dismissed. At this time the MELD score was 16 and the INR 1.6. The corresponding CE-MELD showed 14 points. This case illustrates impressively the possible disturbance of the early conventional MELD score in the context of anticoagulation.
